# Post‐Traumatic Stress in Caregivers to Children and Young People With Eating Disorder (ED) Symptoms: A Cross‐Sectional Examination of Relationships With Demographics, ED Factors and Caregiver Skills

**DOI:** 10.1002/erv.70015

**Published:** 2025-07-29

**Authors:** Natasha Heal‐Cohen, Rachel Nabirinde, Aaron Burgess

**Affiliations:** ^1^ Trainee Clinical Psychologist Cambridgeshire and Peterborough NHS Foundation Trust Department of Clinical Psychology and Psychological Therapies Norwich Medical School University of East Anglia Fulbourn UK; ^2^ Trainee Clinical Psychologist Cambridgeshire and Peterborough NHS Foundation Trust Department of Clinical Psychology and Psychological Therapies Norwich Medical School University of East Anglia Norwich UK; ^3^ Department of Clinical Psychology and Psychological Therapies Norwich Medical School University of East Anglia Norwich UK

**Keywords:** caregiver skills, caregivers, children and young people, eating disorders, post‐traumatic stress

## Abstract

**Objective:**

Post‐traumatic stress disorder (PTSD) symptoms in caregivers of children and young people (CYP) with eating disorder (ED) symptoms remain understudied, despite their potential impact. This study examines these symptoms and their relationship to demographic and ED‐related factors, and caregiver skills. This aims to inform efforts to improve caregivers' wellbeing and ability to support CYP with EDs.

**Method:**

UK‐based parental caregivers of CYP with ED symptoms were recruited via social media and mental health organizations. A total of 123 participants provided demographic and ED‐related information and completed measures of caregiver skills and PTSD symptoms via an online survey. Descriptive statistics, correlations and regressions were conducted.

**Results:**

The majority of participants (62.6%) exhibited scores indicative of probable PTSD. Demographic and ED‐related factors explained 21% of the variance in caregiver PTSD symptoms, with ED relapse contributing the largest independent effect. PTSD symptoms explained 34% of the variance in self‐reported caregiver skills.

**Conclusions:**

Caregivers to a wider ED population than previously studied may be at high risk of PTSD, and symptoms may hinder caregivers' ability to support their child. The link between ED relapse and caregiver PTSD warrants further investigation. Trauma‐informed approaches to caregiver support in child and adult ED services are recommended.

## Highlights

- The majority of parental caregivers (62.6%) of children and young people (CYP) with ED symptoms exhibited scores on a validated measure indicative of probable PTSD.
- Demographic and ED‐related factors together explained a moderate amount of the variance in PTSD symptoms, with small independent associations observed for most variables (including CYP age and gender) and a moderate association found for ED relapse.
- PTSD symptoms accounted for a large portion of the variance across self‐reported caregiver skills such as self‐care and emotional intelligence.

## 1 Introduction

Eating disorders (EDs) are mental health conditions characterized by disturbances in eating behaviours and related thoughts and emotions (American Psychiatric Association 2013). EDs typically onset in adolescence and early adulthood (Wood et al. 2019), however, a trend of decreasing peak age of onset and increasing incidence among prepubertal children has also been observed (Morris et al. 2022; Van Eeden et al. 2021). Nearly all first‐time cases occur by the age of 25 (Ward et al. 2019). They can result in reduced quality of life, significant physical health complications, and mortality (Santomauro et al. 2021; van Hoeken and Hoek 2020). A meta‐analysis by Arcelus et al. (2011) found individuals with anorexia nervosa (AN) had a nearly sixfold times increased risk of mortality (standardised mortality ratio = 5.86).

### 1.1 The Impact of EDs on Caregivers

For children and young people with EDs, the role of parents and carers is particularly crucial, with family‐based interventions regarded as best practice (Gorrell et al. 2019; Le Grange et al. 2010). However, EDs place considerable strain on families, with caregiver burden reported to be higher with EDs than with depression or schizophrenia (Martín et al. 2011; Svensson et al. 20 13). Parents may witness their child's physical deterioration, worrying behaviours which can threaten their health and development (e.g., purging, dietary restriction), and extreme personality changes (Peebles and Sieke 2019; J. Whitney et al. 2005). Common responses of caregivers include denial, self‐blame, anger, helplessness as well as heightened worry and vigilance which persists beyond their child's recovery (Coelho et al. 2021; Fletcher et al. 2021; Fox et al. 2017).

### 1.2 Caregiver Post‐Traumatic Stress

In the UK, around 4% of adults are estimated to have post‐traumatic stress disorder (PTSD) (McManus et al. 2016), although rates are found to be significantly higher among caregivers of offspring with serious physical and psychiatric illnesses, such as cancer or schizophrenia (Carmassi et al. 2021; Kageyama and Solomon 2018; Woolf et al. 2016). PTSD is a mental health condition which can occur following direct or indirect exposure to a stressor and can persist without treatment (American Psychiatric Association 2013). Symptoms include intrusions (e.g., flashbacks), avoidance of trauma reminders, negative alterations in cognitions and mood (e.g., guilt, anger) and alterations in arousal and reactivity (e.g., hypervigilance) (American Psychiatric Association 2013). Symptoms can significantly impair functioning even at subclinical levels (Regel and Joseph 2017).

Despite an emphasis on sudden or acute life‐endangering traumatic events within diagnostic criteria (American Psychiatric Association 2013) caregivers are thought to experience multiple or prolonged traumas in relation to their child's illness with repeated exposure to re‐traumatizing events exacerbating symptoms (Carmassi et al. 2021). The paediatric medical traumatic stress model (Kazak et al. 2006) outlines how stress responses may be normative and adaptive to the ongoing threat of harm to their child, however, when extreme, distressing and persistent they can constitute PTSD. Despite the duty of health services to support caregivers (National Institute for Health and Care Excellence [NICE], 2020), evidence for the effectiveness of interventions to reduce caregiver PTSD is limited (Cherak et al. 2021).

To the authors' knowledge, only three studies have measured post‐traumatic stress in parents of those with EDs, and only in relation to anorexia nervosa (AN). They indicated elevated levels of probable PTSD (Hazell et al. 2014; Irish et al. 2024) and subclinical post‐traumatic stress (Timko et al. 2023). Mothers were found to experience numerous DSM‐IV criteria traumatic stressors throughout their daughter's AN, such as waiting to receive urgent support, being unable to prevent her deterioration, and thinking she was about to die (Hazell et al. 2014).

### 1.3 Factors Related to Post‐Traumatic Stress

Within the cognitive model of PTSD (Ehlers and Clark 2000), the ways in which a person processes the traumatic event and its aftermath (their negative appraisals, coping styles and memory encoding) are considered to underpin the disorder. However, demographic and illness‐related risk factors for PTSD have also been identified in caregivers in response to paediatric illness such as caregiver gender, child age, recent diagnosis, relapses, and lack of support, although evidence is often mixed (Burgess et al. 2021; Carmassi et al. 2021; Woolf et al. 2016).

In relation to ED caregivers, only a limited range of variables have been measured with mixed evidence of associations between PTSD symptoms and parent gender, ED duration and physical markers of ED severity (Irish et al. 2024; Timko et al. 2023) and only in relation to narrowed samples of those with AN. Other factors such as child gender, relapse, diagnosis, and transdiagnostic behaviours (e.g., purging) have yet to be investigated despite associations with ED caregiver distress and different caregiving experiences (e.g., Sepulveda et al. 2014; J. L. Whitney et al. 2023). Better understanding demographic and ED‐related factors associated with caregiver PTSD symptoms is important in improving the identification and targeting of support.

### 1.4 Caregiver Post‐Traumatic Stress and the Caregiving Role

The potential for post‐traumatic stress to impact parents' ability to support their child's recovery is well documented (Carmassi et al. 2021), with initial evidence linking post‐traumatic stress in ED caregivers to greater accommodation of the ED and slower weight gain for their child during recovery (Timko et al. 2023). Interpersonal models emphasize the role of caregivers' emotional and behavioural responses in maintaining EDs (Schmidt and Treasure 2006; Treasure and Schmidt 2013). In light of this, there is growing recognition of the importance of caregiver skills such as emotional intelligence, acceptance, and maintaining hope, in fostering recovery (Langley et al. 2018; Treasure et al. 2016). Research shows that higher caregiver‐reported skill levels and improvements following interventions are associated with better ED outcomes (Magill et al. 2016; Philipp et al. 2021; Salerno et al. 2016). Understanding the relationship between caregiver PTSD symptoms and these skills is crucial for informing interventions that strengthen caregivers' ability to support their child effectively.

### 1.5 Aims

In summary, this study aims to better understand parental caregivers' PTSD symptoms and their relation to demographic, ED‐related factors and the caregiving role, in order to help improve ED caregivers' wellbeing and their ability to support their child. It also aims to extend previous research by exploring PTSD symptoms in a sample of parental caregivers to children and young people (CYP) with a broader range of ED presentations and support experiences. Given the limited prior research in this area, this study adopted an exploratory approach, aiming to generate hypotheses to guide future research. Specifically, the study research questions are: (RQ1) *What is the rate of probable PTSD in the sample as indicated by a validated measure?* (RQ2), *What demographic and ED‐related factors are associated with caregiver PTSD symptoms?* and (RQ3) *What is the relationship between PTSD symptoms and caregiver skills?*

## 2 Method

### 2.1 Participants

Individuals eligible for the study were: (a) parents or guardians to a CYP between 5 and 25 years old currently experiencing eating‐related difficulties due to a suspected or diagnosed ED (of any type), (b) who have a current substantial role in supporting the CYP emotionally and/or practically with their eating‐related difficulties, (c) aged 18 years or older (d) live in the UK and (e) are proficient in English. Given the online format of the study, individuals also needed access to an internet connected device.

This study received UEA Faculty of Medicine and Health Sciences Research Ethics Committee approval (ETH2324‐1470) and conforms to the *British Psychological Society Code of Human Research Ethics* (2021).

Between January and June 2024, 123 caregivers participated in the study. Participants were recruited through voluntary response sampling, with recruitment advertisements shared on social media platforms (Facebook, Instagram, X) and distributed by 13 UK‐wide ED and mental health organizations. Participants were also invited to share the survey link with others. Recruitment efforts emphasized participation from underrepresented groups within adverts and by engaging a diverse range of organizations.

### 2.2 Design

This study utilized a cross‐sectional research design and self‐report online survey methodology. It has been reported in accordance with the STROBE checklist for cross‐sectional studies (STROBE 2023).

### 2.3 Measures

#### 2.3.1 Demographic and ED Information

Participants self‐reported demographic information about themselves and their child, and clinical and support‐related factors associated with their child's ED that are known to be relevant in ED and PTSD fields. This included identifying common ED‐related behaviours (i.e., restriction, binging, vomiting, laxative use, excessive exercise) in their child (Accurso and Waller 2021). Respondents selected answers from pre‐defined options, with a free‐text box provided for those who selected “other”.

#### 2.3.2 Caregiver PTSD Symptoms

The Posttraumatic Stress Disorder Checklist for DSM‐5 (PCL‐5; Weathers et al. 2013) is a standardised self‐report scale comprising of 20 items designed to assess symptoms of PTSD. Respondents rate the severity of each symptom over the past month on a five‐point scale from “not at all” to “extremely”. It has four subscales corresponding to the Diagnostic and Statistical Manual of Mental Disorders, fifth edition (DSM‐5; American Psychiatric Association 2013) symptom criteria: intrusions (Criterion B), avoidance of trauma reminders (Criterion C), negative alterations in cognitions and mood (Criterion D), and alterations in arousal and reactivity (Criterion E). Total scores (0–80) indicate symptom severity, with higher scores denoting higher symptom levels. Cut‐off scores of 31–33 are suggestive of probable PTSD (Forkus et al. 2023), but do not constitute a formal diagnosis, which requires a comprehensive clinical assessment. A cut‐off score of 33 was adopted in this study, consistent with recent ED caregiver research (Irish et al. 2024).

The PCL‐5 is one of the most widely used scales for assessing PTSD symptoms with robust psychometric properties (Forkus et al. 2023), including high internal consistency (Cronbach's *α* > 0.80) in comparable parental populations (Irish et al. 2024; Stewart et al. 2020).

In this study, parents were instructed to respond to questions considering any stressful experience(s) they have had in relation to their child's ED. Cronbach's alpha was 0.93.

#### 2.3.3 Caregiver Skills

The Caregiver Skills Scale (CASK; Hibbs et al. 2015) is used to measure caregiving behaviours toward individuals with an ED. Respondents self‐assess skill level on 27 items from 0 to 100, with higher values indicating more adaptive behaviour. It is based upon the interpersonal mechanisms theorised to maintain EDs (Schmidt and Treasure 2006; Treasure and Schmidt 2013). The scale consists of six subscales reflecting different caregiver skills (see Table 1). CASK total score (0–2700) and separate subscale scores are derived. Cronbach's *α* is high (0.92 for total, 0.71–0.85 for subscales), with good convergent validity and sensitivity to change (Hibbs et al. 2015; Hodsoll et al. 2017). In the present study Cronbach's alpha was 0.93 for the total scale and between 0.70 and 0.86 for subscales.

**TABLE 1:** The six caregiver skills.

Caregiver skill	Definition (from Hibbs et al. 2015)
Bigger picture	The ability to be more positive about changes with a hopeful long‐term view
Self‐care	The ability to take time for self and other family members
Biting your tongue	The ability to control the urge to enquire and avoid repetitive nagging conversations
Insight and acceptance	The ability to accept and manage negative emotions
Emotional intelligence	The ability to discuss and manage feelings
Frustration tolerance	The ability to sidestep conflict yet be firm, calm and understanding toward the person with the ED

### 2.4 Procedure

Participants followed a weblink from the advert to the survey webpage on the platform Jisc Online Surveys (https://www.onlinesurveys.ac.uk). Participants who indicated they met eligibility criteria were then presented with the participant information and gave informed consent by indicating their agreement to all consent statements. Consenting participants then completed the survey (approximately 15 min), which included the demographics and ED information section, PCL‐5, and CASK scale. The survey also included bot‐screening questions (e.g., “which of these is a color?”) and an additional measure that was analysed as part of a separate study. Participants were required to answer all questions to proceed ensuring no missing data, and responses were saved upon completion. Details of how to access mental health and caregiver support were provided in the participant information and debrief pages. As a token of appreciation for participation, a donation of £2 per participant (£246 total) was made to an ED charity, chosen by a local National Health Service (NHS) carer group.

### 2.5 Analysis

Data were analysed using IBM SPSS Statistics (Version 28). Scrutinisation of individual responses identified no missing data, suspect responses, or multiple entries relating to the same CYP. All outliers were reviewed and considered to represent meaningful data. No responses were therefore excluded.

#### 2.5.1 Descriptive Statistics

Descriptive statistics were calculated, with means and standard deviations reported for continuous variables, and frequencies and proportions for categorical variables. To enhance interpretability, some related or small subgroup categories were merged (ethnicity, purging, hospitalization and non‐degree qualifications).

*RQ1.* The proportion of the sample with probable PTSD was calculated based on total PCL‐5 scores using a cut off of 33. The proportion endorsing each symptom criterion was also calculated based on item ratings of “moderately” or higher for at least one item for both Criteria B and C and at least two items for both Criteria D and E as recommended by PCL‐5 developers (National Centre for PTSD 2023).

#### 2.5.2 Inferential Statistics

An a priori power analysis conducted using G*Power 3.1 (Faul et al. 2009) determined that a sample size of 84 participants was required to detect a moderate effect size (*r* = 0.30) with a statistical power of 0.8 and a significance level of *p* < 0.05 (two‐tailed) for the primary correlational analyses. All inferential analyses used a significance level of 0.05, with no statistical corrections for multiple comparisons due to the exploratory nature of the study. Cohen's (1988) benchmarks for small, medium/moderate and large effects were followed (*r* = 0.10, 0.30, 0.50; *R* ^2^ = 0.02, 0.13, 0.26; *η*_p_^2^ = 0.01, 0.06, 0.14). Key assumptions for each analysis were evaluated using visual inspections and diagnostic tests. In cases where deviations from normality or potential outliers were identified, non‐parametric correlations or bias‐corrected bootstrapping (BCa; 1000 samples) was employed to improve robustness. In all other cases, all assumptions were satisfied.

*RQ2*. Correlations were conducted to examine bivariate relationships between PTSD symptom severity (PCL‐5 total score) and demographic and ED‐related variables. To facilitate analysis, CYP support was dichotomized into meaningful groups (treatment ongoing = 1, other categories = 0), and CYP gender was dichotomized (female = 1, male/non‐binary = 0) due to the small non‐binary gender sample size. For multi‐response categories (caregiver support, ED behaviours, parent‐reported ED), each response option was treated as a separate binary (yes/no) variable. Point‐biserial correlations were conducted for binary variables, with bootstrapping to correct for outliers and non‐normality in small groups. Analyses were not conducted for groups with ≤ 5 participants due to validity concerns (Bonett 2020).

A hierarchical multiple regression using the entry method was used to assess the predictive value of demographic and ED‐related variables on PTSD symptom severity (outcome variable). Demographics were entered into the model in the first block and followed by ED‐related variables in the second block. To prevent overfitting, prioritize the most impactful predictors, and ensure adequate statistical power (up to 11 variables), variables were selected based on correlational analyses and theoretical relevance informed by prior research. A bootstrap, applied to check the robustness of the model (Field and Wilcox 2017), did not alter findings and therefore the standard regression is reported. A sensitivity analysis was also carried out to assess the impact of separating male and non‐binary CYP gender using dummy coding with female gender as the reference group.

*RQ3.* Initial correlations were conducted between PTSD symptom severity and caregiver skills (individual and overall). A multivariate multiple regression (Wilk's Lambda multivariate criterion) was then performed to assess the variance in each caregiver skill (outcome variables) explained by PTSD symptoms (predictor variable), whilst accounting for intercorrelations between caregiver skills. Bootstrapping was applied to account for non‐normality of residuals for two caregiver skills.

## 3 Results

### 3.1 Sample Characteristics

Characteristics of caregivers, their child and ED factors are summarized in Table 2. Participants were aged between 28 and 63 (*M* = 50.08, SD = 6.41). Most were mothers (92.7%), identified as white (97.6%), with a university degree (74.8%). Participants cared for CYP aged between 5 and 25 years (*M* = 16.6, SD = 3.8), who were mostly female (87.0%), with a formally diagnosed ED (86.2%). Just under two thirds (63.4%) were currently receiving some form of treatment.

**TABLE 2:** Demographic and ED‐related descriptive statistics and correlations with PTSD symptoms.

	Caregivers (*N* = 123)		Correlation coefficient	*p*‐value
	n/M	%/SD (range)	*r/r*_*s*_ */r*_*pb*_ [BCa CI]	
Caregiver demographics				
Age (years)	50.08	6.41 (28–63)	0.05^b^	0.57
Ethnicity			—	
White	120	97.6%		
Other	3	2.4%		
Highest level of education			0.01^b^	0.89
Non‐degree level (e.g., high school, diploma)	31	25.2%		
Bachelor's degree	42	34.1%		
Master's degree	42	34.1%		
PhD	8	6.5%		
Relationship to CYP (mother = 1)			−0.09 [−0.30, 0.12]	0.32
Mother	114	92.7%		
Father	9	7.3%		
Cohabitation with CYP			0.08^b^	0.37
Full time	92	74.8%		
Most of the time (e.g., 5 days a week)	7	5.7%		
Some of the time (e.g., holidays, weekends)	20	16.3%		
None of the time	4	3.3%		
CYP demographics				
Age (years)	16.61	3.82 (5–25)	0.11^b^	0.22
Gender (female = 1)			−0.13 [−0.27, 0.04]	0.17
Female	107	87.0%		
Male	14	11.0%		
Non‐binary	2	2.0%		
ED‐related factors				
Formally diagnosed ED (yes = 1)			0.05 [−0.13, 0.24]	0.58
Yes	106	86.2%		
No	17^c^	13.8%		
Parent‐reported ED^d^				
AN	98	79.7%	0.05 [−0.13, 0.20]	0.61
BN	7	5.7%	0.18^a^ [0.07, 0.30]	< 0.05
BED	10	8.1%	0.11 [−0.03, 0.25]	0.22
OSFED	3	2.4%	—	
ARFID	23	18.7%	0.08 [−0.11, 0.25]	0.41
Not reported	4	3.3%	—	
ED behaviours^d^				
Restriction	119	96.7%	—	
Excessive exercising	78	63.4%	0.03 [−0.15, 0.20]	0.72
Purging (vomiting or laxative use)	38	30.9%	0.12 [−0.05, 0.29]	0.18
Bingeing	18	14.6%	0.13 [−0.01, 0.27]	0.15
ED duration (years)	4.02	3.51 (0.4–19.9)	0.06^b^	0.53
ED stage (relapse = 1)			0.21^a^[0.05, 0.38]	< 0.05
First episode	67	54.5%		
Relapse	56	45.5%		
Current CYP support (treatment ongoing = 1)			−0.09 [−0.26, 0.10]	0.31
Treatment ongoing	78	63.4%		
Support has ended	22	17.9%		
Awaiting treatment	12	9.8%		
No support available	5	4.1%		
Other	6	4.9%		
Hospitalization (general or psychiatric) (yes = 1)			0.06 [−0.12, 0.25]	0.51
Yes	68	55.3%		
No	55	44.7%		
Caregiver support accessed^d^				
Family‐based therapy	74	60.2%	−0.06 [−0.24, 0.12]	0.49
Support groups	49	39.8%	−0.04 [−0.23, 0.15]	0.64
Individual therapy	48	39.0%	0.08 [−0.09, 0.24]	0.39
Helplines or self‐help resources	81	65.9%	0.05 [−0.13, 0.24]	0.55
None	8	6.5%	−0.04 [−0.20, 0.17]	0.70

*Note*: — correlation not conducted due to group size ≤ 5.

Abbreviations: AN, anorexia nervosa; ARFID, avoidant/restrictive food intake disorder; BCa CI, Bias‐corrected and accelerated bootstrap 95% confidence intervals; BED, binge‐eating disorder; BN, bulimia nervosa; CYP, child or young person; ED, eating disorder; *M*, mean, *n*/N, number of participants; OSFED, other specified feeding and eating disorder; *r*, Pearson's correlation coefficient; *r*_*pb*_, point‐biserial correlation; *r*_*s*_, Spearman's Rho; SD, standard deviation.

^a^ *p* < 0.05.

^b^ *r*_s_ conducted.

^c^ Breakdown of suspected EDs: 11 ARFID, 3 BED, 2 AN, 1 OSFED, 2 Not Reported.

^d^ Categories not mutually exclusive.

### 3.2 PTSD Symptoms (RQ1)

Over half of caregivers (62.6%) exhibited PCL‐5 total scores indicative of probable PTSD (*M* = 38.02, SD = 16.21, range = 2–80). Participants were most likely to endorse symptoms of intrusions (86.2%; Criterion B) and least likely to endorse avoidance of trauma reminders (64.2%; Criterion C).

### 3.3 Demographic and ED‐Related Associations (RQ2)

Correlations between PTSD symptoms and demographic and ED‐related factors are presented in Table 2. Only ED stage and bulimia (BN) showed significant correlations with PTSD symptoms. Both showed small positive correlations indicating that parent‐reported CYP relapse and BN were associated with greater caregiver PTSD symptoms (BN: $r_{pb}$ = 0.18, *p* < 0.05, CI [0.07, 0.31]; Relapse: $r_{pb}$ = 0.21, *p* < 0.05, CI [0.03, 0.38]), although the sample for BN was small (*n* = 7). A post‐hoc power analysis indicated that correlations involving binary groups with distributions exceeding a 25‐to‐98 participant ratio were underpowered (*r* = 0.3, *α* = 0.05, two‐tailed, 80% power).

A hierarchical multiple regression analysis, presented in Table 3, revealed that demographic variables entered in Model 1 (relationship to CYP, CYP gender, CYP age, and cohabitation) did not significantly predict PTSD symptoms, *F* (4, 118) = 2.20, *p* = 0.07, *R* ^*2*^ = 0.07, *adj. R* ^*2*^ = 0.04, although older CYP age was a significant predictor of greater PTSD symptoms. In Model 2, adding ED‐related factors (ED duration, ED stage, purging, bulimia, hospitalization, and current CYP support) significantly improved the model (*ΔR* ^*2*^ = 0.14), with the final model explaining 21% of the variance, *F* (10, 112) = 2.93, *p* < 0.01, *R* ^2^ = 0.21, *adj. R* ^*2*^ = 0.14. Significant predictors of greater PTSD symptoms with small effect sizes (*η*_p_^2^ = 0.04 to 0.05) were male/non‐binary CYP gender, older CYP age, full‐time cohabitation, bulimia, shorter ED duration, and CYP not currently in treatment. CYP relapse was the only variable with a significant moderate‐sized predictive association with PTSD symptoms (*η*_p_^2^ = 0.07).

**TABLE 3:** Regression predicting PTSD symptoms from demographic and ED‐related factors.

Variable	Model 1					Model 2				
		95% CI for B					95% CI for B			
	B	LL	UL	*β*	*η*_p_^2^	B	LL	UL	*β*	*η*_p_^2^
Demographic										
Relationship to CYP (mother = 1)	−6.86	−17.84	4.11	−0.11	0.01	−8.87	−19.50	1.75	−0.14	0.02
CYP gender (female = 1)	−6.70	−15.34	1.94	−0.14	0.02	−10.31	−19.15	−1.48	−0.21*	0.05
CYP age	0.90	0.06	1.74	0.21*	0.04	1.07	0.11	2.03	0.25*	0.04
Cohabitation (full‐time = 1)	6.56	−0.79	13.91	0.18	0.03	9.15	1.98	16.31	0.25*	0.05
ED‐related										
Duration of ED						−1.16	−2.19	−0.14	−0.25*	0.04
ED stage (relapse = 1)						8.81	2.61	15.00	0.27**	0.07
Purging						2.80	−3.67	9.27	0.08	0.01
Bulimia nervosa						13.82	0.89	26.74	0.20*	0.04
Hospitalization						0.97	−4.82	6.75	0.03	0.00
Current CYP support (ongoing treatment = 1)						−6.30	−12.49	−0.11	−0.19*^a^	0.04
*R* ^*2*^	0.07					0.21				
*Adjusted R* ^*2*^	0.04					0.14				
*F‐statistic for change in R* ^*2*^	2.20					3.24**				

Abbreviations: *β*, standardised coefficient; *η*_p_^2^. partial eta squared measure of effect size; *B*, unstandardized coefficient; CI, confidence interval; CYP, child or young person; ED, eating disorder; LL/UL, lower limit/upper limit; *R* ^*2*^, the variance in PTSD symptoms explained by the model.

^a^ Non‐significant (*p* = 0.056) in sensitivity analysis when CYP Gender is dummy coded.

*p < 0.05, **p < 0.01.

All regression assumptions were met, and results remained unchanged after applying a bootstrap procedure, indicating model robustness. A sensitivity analysis assessed the impact of separating male and non‐binary CYP gender using dummy coding with female gender as the reference group. In the re‐run regression, the male dummy variable was significant (*p* < 0.05, *η*_p_^2^ = 0.05), while the non‐binary variable was non‐significant (*p* = 0.82, *η*_p_^2^ < 0.01), likely due to the small sample size (*n* = 2). This suggests that the association between CYP gender and PTSD symptoms is primarily driven by the effect of being male compared to female. No other notable differences were found in the final model, except the small effect of Current CYP support on PTSD symptoms became non‐significant (*p* = 0.056), suggesting this association should be interpreted with caution.

### 3.4 Caregiver Skills Associations (RQ3)

Self‐reported total caregiver skill levels varied considerably across the sample (*M* = 1527.15, SD = 393.39, range = 530–2400). On average, caregivers rated themselves lowest on Self‐Care items (*M* = 42.20, SD = 19.15) and highest on Bigger Picture items (*M* = 65.56, SD = 16.88).

PTSD symptoms showed significant small to large negative correlations with all six caregiver skills and total skill level (−0.27 to −0.52) as summarized in Table 4. A multivariate multiple regression analysis, with bootstrapping applied to account for non‐normality, found PTSD symptoms (predictor) accounted for a significant 34% of the variance across all caregiver skills (outcome variables), Wilks' *Λ* = 0.66, *F* (6, 116) = 10.01, *p* < 0.001, *η* ^*2*^ = 0.34. PTSD symptoms significantly predicted each caregiver skill individually as shown in Table 5, explaining the least variance in Frustration Tolerance (11%) and the most in Self‐Care (26%).

**TABLE 4:** Caregiver skill descriptive statistics and correlations with PTSD symptom severity.

Caregiver skill	Scale range	Total score		Item score		Corr. Coef. *r/r*_*s*_
		M	SD	M	SD	
Total caregiver skills	0–2700	1527.15	393.39	56.56	14.57	−0.52***
Bigger picture	0–700	458.94	118.15	65.56	16.88	−0.40***
Self‐care	0–400	168.78	76.58	42.20	19.15	−0.51***
Biting your tongue	0–300	149.76	55.71	49.92	18.57	−0.43***
Insight and acceptance	0–300	160.65	61.11	53.55	20.37	−0.45***
Emotional intelligence	0–500	264.23	101.92	52.85	20.38	−0.34***
Frustration tolerance	0–500	324.80	91.07	64.96	18.21	−0.27**^a^

Abbreviations: Corr. Coeff, correlation coefficient; M, mean; *r*, Pearson's correlation, *r*_*s*_, Spearman's rho; SD, standard deviation.

^a^ *r*_*s*_ conducted due to non‐normality.

***p < 0.001, **p < 0.01.

**TABLE 5:** Results of regression predicting caregiver skills from PTSD symptoms.

Caregiver skill		95% BCa CI for B			
	B	LL	UL	*F*‐statistic	*R* ^2^
Bigger picture	−2.91	−4.28	−1.57	22.90***	0.16
Self‐care	−2.42	−3.12	−1.70	43.24***	0.26
Biting your tongue	−1.48	−2.01	−0.93	27.59***	0.19
Insight and acceptance	−1.70	−2.30	−1.07	30.72***	0.20
Emotional intelligence	−2.16	−3.12	−1.31	16.25***	0.12
Frustration tolerance	−1.84	−2.68	−0.97	14.48***	0.11

Abbreviations: *B*, unstandardized coefficient; BCa CI, bias‐corrected and accelerated bootstrap confidence interval; LL/UL, lower limit/upper limit; *R* ^*2*^, the variance in caregiver skills explained by PTSD symptoms.

***p < 0.001.

## 4 Discussion

This study investigated post‐traumatic stress in 123 parental caregivers (92.7% mothers, 7.3% fathers) of CYP with ED symptoms. Findings revealed over half (62.6%) exhibited scores indicative of probable PTSD in relation to their child's ED. Demographic and ED‐related factors together explained a moderate amount of the variance in PTSD symptoms, with ED relapse contributing the largest independent association. A number of factors, including CYP age, CYP gender and cohabitation, showed significant relationships with PTSD symptoms only after controlling for the influence of other variables. Additionally, PTSD symptoms accounted for a large portion of the variance in caregiver skills, with greater PTSD symptoms associated with less adaptive caregiving behaviours, such as reduced self‐care, difficulty maintaining hope and increased emotional reactivity in relation to their child's ED.

Findings align with existing evidence of elevated post‐traumatic stress in parents of individuals with AN (Hazell et al. 2014; Timko et al. 2023), with 62.6% of participants exhibiting scores indicative of probable PTSD, comparable to the 56.9% reported in parents of adolescents with AN using the same PCL‐5 cut‐off (Irish et al. 2024). The high proportion with scores suggestive of probable PTSD may be influenced by the impact of acute stress responses to ongoing traumas on PCL‐5 scores and the use voluntary self‐report methods. Additionally, the sample was predominantly composed of mothers. Although the limited number of fathers meant the analysis was underpowered to detect effects of caregivers' relationship to CYP, research suggests female caregivers are at higher risk for developing PTSD (Carmassi et al. 2021).

Importantly, findings of this study indicate that caregivers to a broader ED population than previously identified are at risk of PTSD, including those whose child has no diagnosis, is not accessing support, and presents with avoidant/restrictive food intake disorder (ARFID) or BN. Neither the type of ED nor its formal diagnosis were related to caregiver PTSD symptoms, except for increased levels among parents of children with BN, though the small sample sizes warrant a cautious interpretation.

The finding that some demographic and ED‐related variables showed significant associations with PTSD symptoms only when controlling for others indicates the complex interplay of variables with potential confounding or moderating influences. Combined with their small predictive power, this emphasizes the challenges of identifying caregivers at risk of PTSD based solely on objective characteristics, and the limitations of targeting support toward specific groups.

Some associations, however, are notable. Having a male/non‐binary child with an ED predicted greater caregiver PTSD symptoms, which contrasts the lack of a gender effect found in other fields (e.g., Burgess et al. 2021) and may reflect reported negative impacts of the gender‐atypical nature of EDs on caregivers' experiences, such as challenges accessing support (J. L. Whitney et al. 2023).

The finding that PTSD symptoms were higher when the CYP was not in treatment may reflect caregivers' distress in feeling alone and unsupported whilst awaiting treatment or following discharge (Crowther et al. 2024; Robinson et al. 2020). However, the small effect size and non‐significance in sensitivity analysis suggests any protective effect of treatment on caregiver PTSD is minimal. Similarly, reports of caregivers' feelings of helplessness on being excluded from treatment once their child turned 18 (Crowther et al. 2024) may help to explain higher PTSD symptoms in caregivers of older CYP. The relationship between caregiver PTSD and their experiences engaging with their child's treatment warrants further investigation.

Among the demographic and ED factors examined, child relapse had the largest and most statistically robust association with PTSD, independent of potential confounders such as ED duration, hospitalization, and presentation. This aligns with Burgess et al. (2021) who identified relapse or readmission as a small PTSD risk factor for parents of children with chronic illnesses. Relapse may trigger memories of past trauma related to the ED, intensifying existing symptoms. Caregivers' perceptions that relapse indicates efforts to support their child have failed (Burman et al. 2024) may also play a role as low confidence in treatment has been linked to PTSD in caregivers of cancer patients (Richardson et al. 2016). Conversely, caregiver PTSD symptoms may increase the likelihood of relapse by affecting their ability to support their child's recovery, consistent with longitudinal evidence that caregiver PTSD predicts poorer child outcomes (Bryant et al. 2018; Timko et al. 2023).

Findings support a connection between PTSD symptoms and less adaptive caregiver behaviours that extend beyond accommodation of ED (Timko et al. 2023). Results suggest post‐traumatic stress may have a comprehensive influence across caregiver skills rather than restricted to specific skills and highlight potential mechanisms through which PTSD symptoms may affect ED outcomes. This underscores the relevance of literature on post‐traumatic stress to understanding caregiver responses and extending interpersonal maintenance models of EDs (Schmidt and Treasure 2006; Treasure and Schmidt 2013). While research shows that brief interventions can improve caregiver skills (Miskovic‐Wheatley et al. 2024), without addressing PTSD symptoms such as avoidance and elevated sense of threat, the development of these important skills may be compromised for some caregivers.

### 4.1 Limitations

This study has several limitations. Reliance on self‐report measures carries implications for internal validity. Scoring above cut‐off on the PCL‐5 does not confirm a PTSD diagnosis would be made if assessed by a qualified clinician able to consider factors such as the nature of the trauma, duration and functional impact of symptoms, and differential diagnoses. Similarly, parents' reports of their child's ED are unable to be verified by qualified clinicians, and may contain some inaccuracies, particularly in relation to diagnosis. Additionally, perceived caregiver skills may be influenced by negative affect and self‐concept, and as such, may not reflect caregivers' true abilities.

The study's cross‐sectional design is useful for generating hypotheses but does not allow for causal inferences and as highlighted in relation to relapse, some relationships with PTSD may be bidirectional. The use of multiple comparisons, the potential presence of unmeasured confounders (e.g., body mass index, comorbidities, family composition), and the reduction of data for analytical purposes necessitate cautious interpretation of the results.

Despite recruitment efforts, certain groups (e.g., ethnic minorities, fathers, and caregivers of individuals with non‐restrictive EDs) were underrepresented in the sample, consistent with previous ED research (Halbeisen et al. 2022). This resulted in some unbalanced and small sub‐groups, limiting statistical power to detect effects. Additionally, the study's focus on the UK could restrict wider generalizability due to cultural influences on PTSD (Asnaani and Hall‐Clark 2017). While there was considerable variation in PTSD symptoms in the sample, PTSD‐driven avoidance of their child's ED may have prevented some caregivers from participating. Finally, there is a possibility of unintentional overlap between participants in this study and those in Irish et al. (2024).

### 4.2 Clinical Implications

Findings indicating a high prevalence of probable PTSD in the sample and its association with poorer caregiver skills point to the importance of engaging and supporting caregivers across child and adult ED services. The prevalence and challenges predicting PTSD support the implementation of trauma‐informed care, which emphasizes recognizing trauma's signs and preventing re‐traumatization through system‐level approaches (Office for Health Improvement and Disparities 2022 Health Improvement and Disparities, 2022). Formulations and ED treatment plans would also benefit from considering parental trauma. Support around relapse and trauma‐informed adaptations to caregiver skill interventions may prove valuable. However, whether caregivers can and should be supported within ED services or referred on to specialist services requires consideration given current demands on services.

### 4.3 Further Research

Future research should adopt a proactive approach to increasing sample diversity and share effective recruitment methods for engaging underrepresented populations. Longitudinal studies are needed to understand changes in PTSD over time, causality in relationships with caregiving behaviours and child outcomes such as relapse, and its relative influence compared to other forms of caregiver psychopathology (e.g., depression). Research on caregiver interventions would benefit from assessing their alignment with trauma‐informed principles and routinely including caregiver PTSD outcome measures. Additionally, the suitability and effectiveness of PTSD treatments for ED caregivers, such as trauma‐focused CBT adapted for contexts of ongoing threat (Ennis et al. 2021), warrants investigation.

## Author Contributions

**Natasha Heal‐Cohen:** conceptualisation, investigation, methodology, formal analysis, visualisation, project administration, writing – original draft, writing – review and editing. **Rachel Nabirinde:** conceptualisation, project administration, methodology, investigation. **Aaron Burgess:** conceptualisation, supervision, project administration, methodology, writing – review and editing.

## Ethics Statement

Ethical approval was obtained from UEA Faculty of Medicine and Health Sciences Research Ethics Committee (ETH2324‐1470).

## Consent

Participants provided consent for the publication of study findings based upon the anonymous data they provided.

## Conflicts of Interest

The authors declare no conflicts of interest.

## Data Availability

The anonymous data that support the findings of this study will be made openly available at https://research‐portal.uea.ac.uk/en/datasets/.
